# Differential Effect of Three Macrolide Antibiotics on Cardiac Pathology and Electrophysiology in a Myocardial Infarction Rat Model: Influence on Sodium Nav1.5 Channel Expression

**DOI:** 10.3390/ph14070597

**Published:** 2021-06-22

**Authors:** Noha E. Farag, Mohamed K. El-Kherbetawy, Hussein M. Ismail, Ahmed M. Abdelrady, Eman A. Toraih, Walid Kamal Abdelbasset, Rehab M. Lashine, Mohammed EL-dosoky, Sally Yussef Abed, Khalid M. Ibraheem, Manal S. Fawzy, Sawsan A. Zaitone

**Affiliations:** 1 Department of Physiology, Faculty of Medicine, Suez Canal University, Ismailia 41522, Egypt; nohafarag@gmail.com; 2 Department of Physiology, College of Medicine, Taif University, Taif 21974, Saudi Arabia; 3 Department of Pathology, Faculty of Medicine, Suez Canal University, Ismailia 41522, Egypt; Mohamed_elkherbetawy@med.suez.edu.eg; 4 Department of Cardiology, Faculty of Medicine, Suez Canal University, Ismailia 41522, Egypt; drhussein72@gmail.com; 5 Ministry of Health and Population, Ismailia 41618, Egypt; dr_ahmed_rady73@yahoo.com; 6 Department of Surgery, School of Medicine, Tulane University, New Orleans, LA 70112, USA; etoraih@tulane.edu; 7 Genetics Unit, Histology and Cell Biology Department, Faculty of Medicine, Suez Canal University, Ismailia 41522, Egypt; 8 Department of Health and Rehabilitation Sciences, College of Applied Medical Sciences, Prince Sattam Bin Abdulaziz University, Alkharj 16278, Saudi Arabia; w.kamal@psau.edu.sa; 9 Department of Physical Therapy, Kasr Al-Aini Hospital, Cairo University, Giza 12613, Egypt; 10 Department of Clinical Pharmacology, Faculty of Medicine, Suez Canal University, Ismailia 41522, Egypt; rehab_lashine@yahoo.com; 11 Department of Neuroscience Technology, College of Applied Medical Science in Jubail, Imam Abdulrahman Bin Faisal University, Jubail 35816, Saudi Arabia; mesalama@iau.edu.sa; 12 Department of Respiratory Care, College of Applied Medical Science in Jubail, Imam Abdulrahman Bin Faisal University, Jubail 35816, Saudi Arabia; Syabed@iau.edu.sa; 13 Department of Anesthesia Technology, College of Applied Medical Sciences in Jubail, Imam Abdulrahman Bin Faisal University, Jubail 35816, Saudi Arabia; kmibraheem@iau.edu.sa; 14 Department of Medical Biochemistry and Molecular Biology, Faculty of Medicine, Suez Canal University, Ismailia 41522, Egypt; 15 Department of Biochemistry, Faculty of Medicine, Northern Border University, Arar 1321, Saudi Arabia; 16 Department of Pharmacology & Toxicology, Faculty of Pharmacy, University of Tabuk, Tabuk 714, Saudi Arabia; 17 Department of Pharmacology & Toxicology, Faculty of Pharmacy, Suez Canal University, Ismailia 41522, Egypt

**Keywords:** azithromycin, cardiotoxicity, clarithromycin, ECG, erythromycin, myocardial infarction rat model, Nav1.5

## Abstract

Macrolides were reported to have cardiotoxic effects presented mainly by electrocardiogram (ECG) changes with increased risk in cardiac patients. We aimed to determine the impact of three macrolides, azithromycin, clarithromycin and erythromycin, on cardiac electrophysiology, cardiac enzyme activities, histopathological changes, and sodium voltage-gated alpha subunit 5 (Nav1.5) channel expression. We used eight experimental groups of male albino rats: vehicle, azithromycin (100 mg/kg), clarithromycin (100 mg/kg), erythromycin (100 mg/kg), MI + vehicle, MI + azithromycin (100 mg/kg), MI + clarithromycin (100 mg/kg) and MI + erythromycin (100 mg/kg); each group received chronic oral doses of the vehicle/drugs for seven weeks. ECG abnormalities and elevated serum cardiac enzymes were observed particularly in rats with AMI compared to healthy rats. Microscopic examination revealed elevated pathology scores for rats treated with clarithromycin in both experiments following treatment with erythromycin in healthy rats. Although rats with MI did not show further elevations in fibrosis score on treatment with macrolides, they produced significant fibrosis in healthy rats. Downregulation of cardiac Nav1.5 transcript was observed following macrolides treatment in both groups (healthy rats and rats with MI). In conclusion, the current findings suggested the potential cardiotoxic effects of chronic doses of macrolide antibiotics in rats with MI as manifested by abnormal ECG changes and pathological findings in addition to downregulation of Nav1.5 channels. Furthermore, in the current dose ranges, azithromycin produced the least toxicity compared to clarithromycin and erythromycin.

## 1. Introduction

Macrolides are commonly used antibiotics as they account for 22% of all prescribed antibacterial agents [1]. Although there is a lack of randomized controlled trials that evaluate macrolides use in COVID-19 patients, some studies explored the impact of macrolides combined with hydroxychloroquine [2,3]. Such a combination may increase the risk of QT prolongation, heart failure, and cardiovascular mortality [4]. 

One of the cardiovascular diseases (CVD), acute myocardial infarction (AMI), is among the main reasons for hospital admission and mortality worldwide [5]. Hence, further related research is required to reduce the impact of such devastating disease in line with the world health organization’s (WHO) recommendations to reduce the mortality from non-communicable diseases by 25% for people less than 70 by 2025 [6]. Such a goal can be achieved through more rigorous preventive action plans on the risk factors and mortality decline via increasing treatment access [6]. 

Sudden cardiac death is one of AMI’s main complications that is most probably due to lethal ventricular arrhythmias [2]. This latter outcome accounts for the majority of deaths among other causes of sudden cardiac death, which include cardiomyopathies, channelopathies, and long QT syndrome [3]. MI causes substantial changes in the ion channel function, resulting in post-infarction channel dysfunction that increases action potential duration (APD), impairs repolarization, and predisposes to early afterdepolarizations; hence, ventricular arrhythmias ensue [7]. One of the major players that mediate the above disorders is the cardiac ion channels, including the voltage-gated sodium channels. The most abundant one in the myocardium is Nav1.5 (sodium voltage-gated channel alpha subunit 5). It is composed of an α subunit and a smaller β subunit responsible for “the initial upstroke of the AP in an electrocardiogram (ECG)”. It is regulated by many factors, including intracellular and extracellular proteins, calcium ions, drugs, and the cytoskeleton’s mechanical properties [8]. The myocardial cells rely on the fast sodium current (stage 0 of cardiac muscle depolarization) that causes a rapid influx of sodium (INa) which results in depolarization of myocardial cells and impulse conduction [9]. A growing interest in Nav1.5 and its alterations due to many pathological conditions as long QT syndrome, atrial fibrillation, Brugada syndrome, and arrhythmogenic ventricular dysplasia was observed [8].

Many medications are known to prolong ventricular repolarization and QT interval, so they potentially cause torsade de pointes. These medications include some antibiotics (for instance, macrolides) [10]. Though they are generally well-tolerated, they retain potentially side effects such as QT prolongation [11]. Although azithromycin increases cardiac conduction, adverse cardiovascular outcomes are mainly found in patients with cardiovascular diseases, including MI [12].

We designed the current experiment to elucidate the effect of three different macrolides and estimate their adverse effects on MI rats compared to healthy rats. Hence, we can expand our expectation towards their impact on humans with a history of acute MI, whether it will provide the same degree of toxicity or an exaggerated outcome. Additionally, we tested the possible effect on the expression of the cardiac sodium (Nav1.5) channel that was not previously excluded or proven in MI models.

## 2. Results

### 2.1. The Effect of Azithromycin, Clarithromycin, and Erythromycin on Serum Lactate Dehydrogenase and CK-MB in Healthy Rats and AMI Rats 

**Figure 1:**
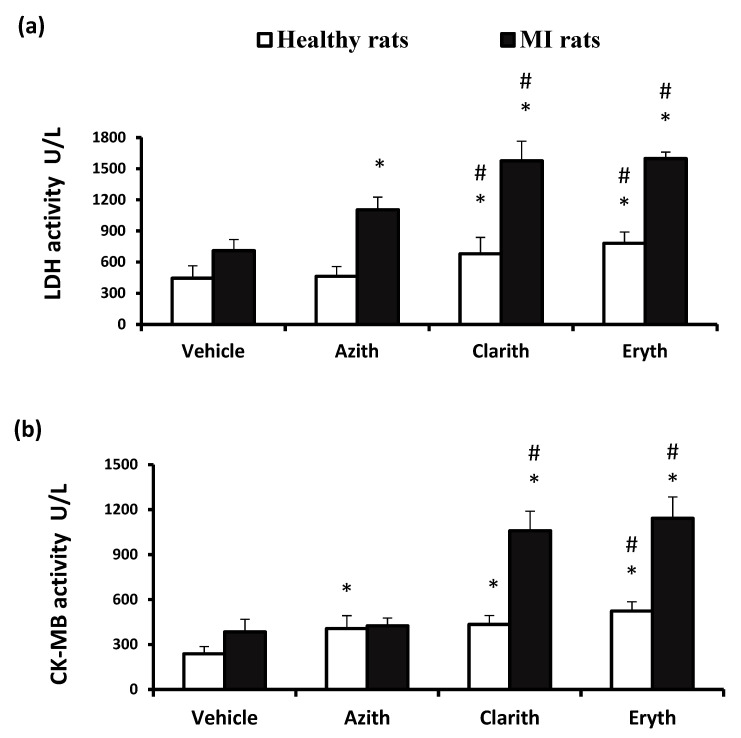
Serum cardiac enzyme activities in the rat experiments. (**a**) LDH activity and (**b**) CK-MB activity in healthy or MI rats treated with azithromycin (Azith), clarithromycin (Clarith), or erythromycin (Eryth). MI: myocardial infarction, LDH: lactate dehydrogenase, CK-MB: creatinine kinase—myocardial band. Data in each set were presented as mean ± standard deviation, and analyzed separately by one-way ANOVA and Bonferroni’s test. * Different from the vehicle group at *p*-value less than 0.05. # Different from the azithromycin group of the same experiment at *p*-value less than 0.05 (*n* = 4–6).

The serum cardiac enzyme levels are presented in Figure 1. Healthy rats treated with azithromycin (Azith) did not show increased LDH activity; however, rats treated with clarithromycin (Clarith) or erythromycin (Eryth) showed elevated LDH levels compared to the vehicle treated healthy group and the Azith treated group (Figure 1a). The serum CK-MB level was found raised in the three antibiotic groups versus the vehicle treated healthy group. Importantly, the CK-MB levels in Clarith and Eryth groups were greater than the Azith group (Figure 1b). Regarding the MI experiment, the MI control rats showed greater LDH and CK-MB activities versus the vehicle group in healthy rats (Figure 1a,b).

Treatment with any three antibiotics produced significant elevations in serum LDH level. Further, the enzyme level in MI + Eryth groups was significantly higher than the MI + Azith group (Figure 1a). Serum CK-MB level was elevated in MI + Clarith and MI + Eryth versus the MI control group and the MI + Azith group (Figure 1b). 

### 2.2. ECG Parameters in Normal and AMI raTs Received Azithromycin, Clarithromycin, and Erythromycin

**Figure 2:**
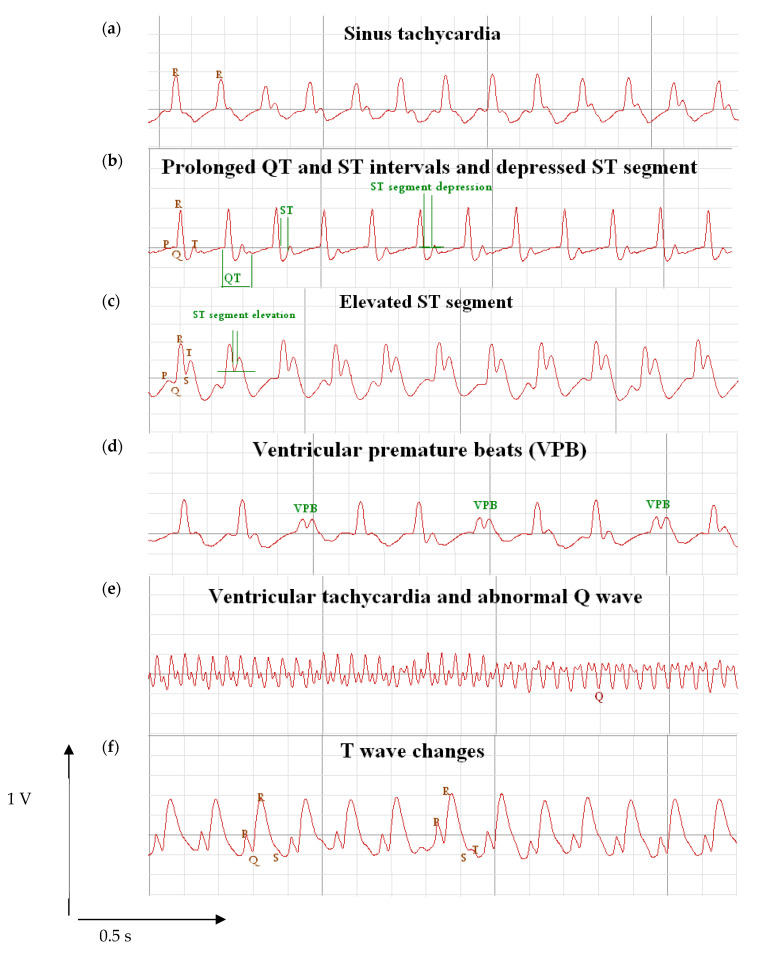
Electrocardiograms from rats with isoproterenol hydrochloride treated group. Rats were subjected to ECG recording under ether anesthesia in the Biopac data acquisition mp150 device. The figure represents electrocardiograms for rats with myocardial infarction group showing (**a**) tachycardia, (**b**) QT prolongation and depressed ST, (**c**) ST-segment elevation, (**d**) ventricular premature beats, (**e**) abnormal Q wave, and (**f**) T wave changes.

Figure 2 demonstrates some important findings that were observed in the MI rats in the current study. Panel 2a displays a trace of a rat showing sinus tachycardia. Panel 2b displays prolonged QT segment and ST-segment plus ST-segment depression, whereas Panel 2c shows ST-segment depression. Also, Panel 2d & 2e show ventricular premature beats and ventricular tachycardia, respectively. Finally, Panel F shows examples of T wave changes.

**Figure 3:**
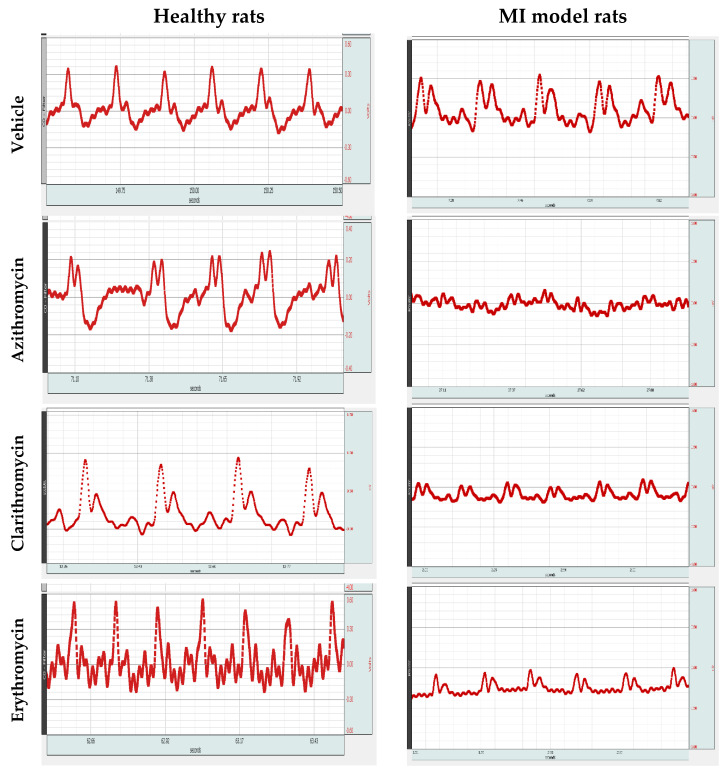
Electrocardiograms from healthy or myocardial infarction model rats treated with Azithromycin, Clarithromycin, and Erythromycin. Rats were subjected to ECG recording under ether anesthesia in the Biopac data acquisition mp150 device. The left side images for healthy rats treated with azithromycin, clarithromycin or erythromycin showing ST-segment elevation and QT prolongation. The right side images are ECGs from the myocardial infarction rat model experiment treated with azithromycin, clarithromycin or erythromycin.

Figure 3 illustrates ECG traces from the healthy rats and MI model rats treated with similar doses of each antibiotic. The healthy rat panel shows abnormal ECG trace with QT interval prolongation upon treatment with the three macrolide antibiotics. 

On the other hand, the MI model rat panel demonstrates atypical ECG shape demonstrating most of MI features such as tachycardia (~5% increase in the MI + vehicle group more than the healthy vehicle group), ST-segment elevation, ventricular premature beats, abnormal Q wave and T wave changes. 

The effect of treatment with Azith, Clarith and Eryth was seen mainly in form of QT interval prolongation. For example, in Clarith + MI and Eryth + MI groups, there were significantly higher values that reached ~5% increase in QT interval more than the MI + vehicle group. In addition, ventricular premature beats which are seen very clearly in MI + Azith and MI + Clarith goups. Further, the HR increased in all the MI model rats received the macrolide antibiotics; for example, MI + Eryth and MI + Clarith groups showed significant (~10%) increases in HR compared to the MI + vehicle group (Figure 3). 

Table 1 demonstrates the ECG parameters in the healthy rats; the PR interval was prolonged in Azith, Clarith, and Eryth groups versus the vehicle treated healthy group. Also, the QT interval and HR values in the three antibiotic groups were elevated versus the vehicle treated healthy group.

The ECG parameters for the MI model rats are shown in Table 1. The PR, the QT and the HR in the MI control group were greater than the vehicle group in healthy rats. The PR and the QT intervals were prolonged with tachycardia in the MI + Clarith and MI + Eryth groups compared to the MI control rats (Table 1). 

### 2.3. Effect of Azithromycin, Clarithromycin, and Erythromycin on Myocardial Histopathologic Findings 

**Figure 4:**
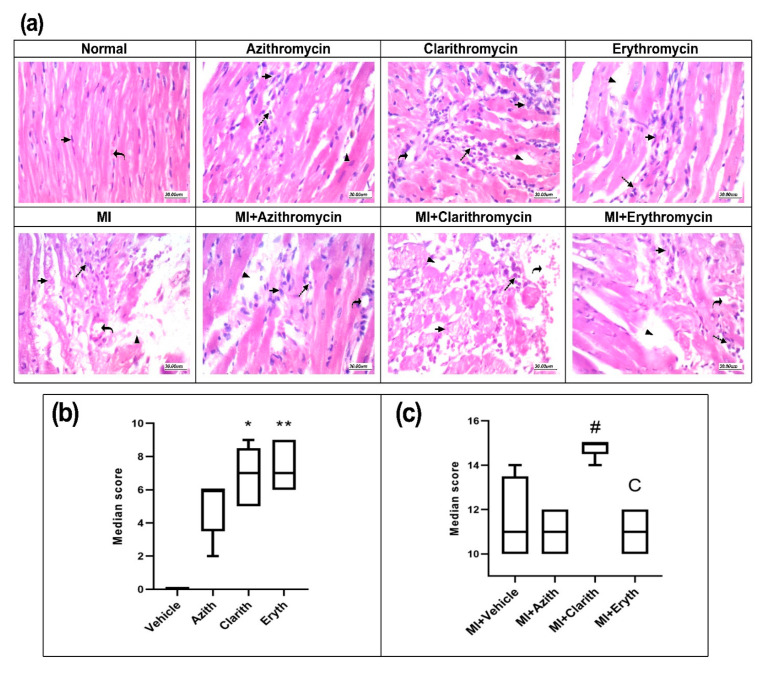
Hematoxylin and eosin staining for cardiac specimens in the two rat experiments. (**a**) Images for specimens were taken from healthy or MI rats treated with azithromycin (Azith), clarithromycin (Clarith), or erythromycin (Eryth). (**b**) Histologic scores for healthy rat experiment and (**c**) Histologic scores for MI rat experiment. MI: myocardial infarction. Data presented as medians, and interquartile ranges for the histologic score in each set, were analyzed separately by Kruskal-Wallis ANOVA and Dunn’s Test. * *p*-value less than 0.05, ** *p*-value less than 0.01 different from the vehicle group. # *p*-value less than 0.05: different from the azithromycin group of the same experiment. ^C^*p*-value less than 0.05: different from the MI + Clarith group (*n* = 4–6).

Figure 4a shows cardiac tissues stained with HE from the experimental groups. Some pathologies were observed and assessed according to a documented criterion. Figure 4 demonstrates the Hx&E cumulative score in experiment 1 and indicates that the cumulative score recorded in the Clarith and Eryth groups was higher than the cumulative score reported in the vehicle group (Figure 4b). In experiment 2, the MI control group showed greater histology score compared to the vehicle group in healthy rats. The MI + Clarith group was the sole group which presented a higher score versus the MI control group (Figure 4c).

**Figure 5:**
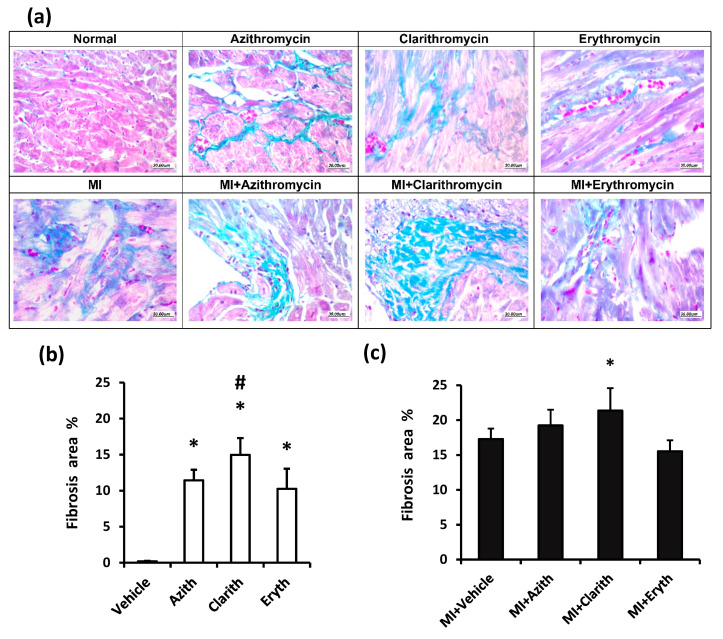
Masson’s trichrome staining for cardiac specimens in the two rat experiments. (**a**) Images for specimens were taken from healthy or MI rats treated with azithromycin (Azith), clarithromycin (Clarith), or erythromycin (Eryth). (**b**) Fibrosis % for healthy rat experiment and (**c**) Fibrosis % for MI rat experiment. MI: myocardial infarction. Data are medan ± standard deviation for each set’s histologic score analyzed separately by one-way ANOVA and Bonferroni’s test. * *p*-value less than 0.05, different from the vehicle group. # *p*-value less than 0.05: different from the azithromycin group of the same experiment (*n* = 4–6).

Masson’s trichrome staining in the study groups is shown in Figure 5a. Cardiac specimens show different degrees of blue staining, which is indicative of fibrosis. The fibrosis area in experiment 1 is shown in Figure 5b and reveals that all the antibiotic groups showed greater fibrosis than the vehicle group. The fibrotic area in the Clarith group was higher compared to that registered in Azith treated rats. In Figure 5c, the MI control group displayed a wider fibrosis area compared to the vehicle group in healthy rats. The fibrosis area in the MI + Clarith group was greater than the MI control group (Figure 5c).

### 2.4. The Survival Percent in Experimental Groups 

**Figure 6:**
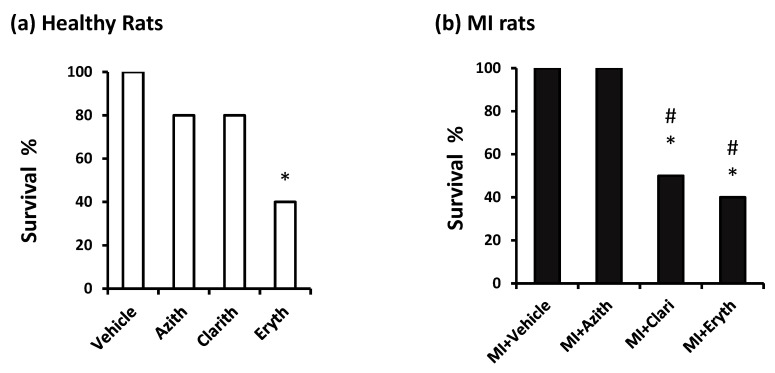
Survival percent in the two rat experiments. (**a**) healthy rats, and (**b**) acute MI rats. Survival % was calculated in normal or MI rats treated with azithromycin (Azith), clarithromycin (Clarith), or erythromycin (Eryth). MI: myocardial infarction. Data in each set were analyzed separately by Chi-square Test. Data are % out of 10 rats. * Different from the vehicle group at *p* less than 0.05. # Different from the azithromycin (Azith) group of the same experiment at *p*-value less than 0.05.

Figure 6 demonstrates the survival % in the study groups. In experiment 1: Vehicle treated healthy rats showed 100% survival until the end of the experiment. Azith (80%) and Clarith (80%) groups did not demonstrate a significantly lower survival % versus the vehicle treated healthy rats; however, the Eryth group showed a significantly lower survival % (40%) than the vehicle control rats (Figure 6a). In experiment 2: the MI control rats and MI + Azith rats showed 100% survival whereas, MI + Clarith and MI + Eryth groups showed significantly lower survival % compared to the MI control group (50% & 40%, respectively, Figure 6b).Notably, the MI control group did not show a higher mortality % in comparison to the vehicle group in healthy rats.

### 2.5. The Effect of Azithromycin, Clarithromycin, and Erythromycin on Cardiac Nav1.5 Channel Gene Expression 

**Figure 7:**
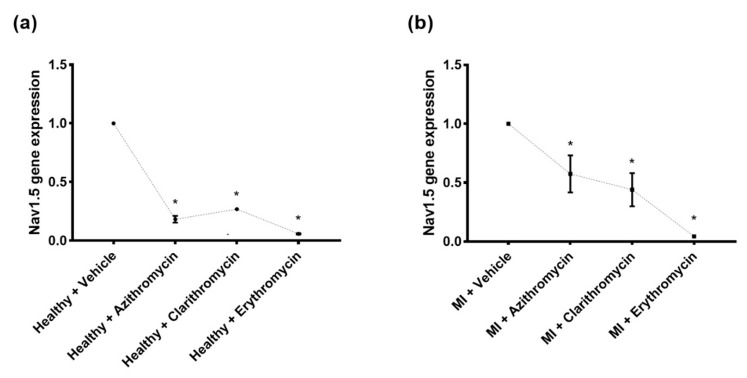
Cardiac *SCN5A* gene expression analysis in the study rat groups. Glyceraldehyde-3-phosphate dehydrogenase (*GAPDH)* was applied as an endogenous control in Real-Time PCR runs for data normalization. Relative quantification of the *SCN5A* gene expression was done based on the quantitative cycle (Cq) values generated from the PCR with the equation: 2^−ΔΔCq^ where ΔΔC_q_ (delta delta quantitative cycle) = (C_q_ *SCN5A* − C_q_ *GAPDH*)_MI group_ − (C_q_ *SCN5A* − C_q_ *GAPDH*)_Healthy control_ [13]. (**a**). Normal rats were subjected to azithromycin, clarithromycin, and erythromycin (200 mg/kg for each drug) versus normal rats without treatment (healthy control). (**b**). Rats with induced myocardial infarction (MI) subjected to treatment with the same antibiotics versus the MI control group. The mean value of the controls in each group was set at value 1. * Significantly different from saline group in panel (**a**) (*p*-values < 0.001), * Significantly different from MI group in panel (**b**) (*p*-values < 0.001).

Figure 7 shows the relative cardiac Nav1.5 channel gene expression in the different study groups quantified by quantitative reverse transcription-polymerase chain reaction (qRT-PCR) after treatment with the three specified antibiotics. The mean expressions of the studied cardiac channel in the untreated groups were set at value “one” according to the applied Livak method (explained in the Section 4.9. First, in healthy rats, *SCN5A* gene relative expression was reduced in those treated with Azith [median (Q1–Q3): 0.183 (0.097–0.270)], Clarith [median (Q1–Q3): 0.269 (0.253–0.286)], or Eryth [median (Q1–Q3): 0.060 (0.044–0.076)] relative to the vehicle treated healthy group (Figure 7a). Second, in the MI rat model experiment, significant reductions were also observed in the relative myocardial Nav1.5 expression after treatment with any of the three macrolide antibiotics in MI + Azith [median (Q1–Q3): 0.574 (0.102–1.046)], MI + Clarith [median (Q1–Q3): 0.440 (0.017–0.863)], and MI + Eryth [median (Q1–Q3): 0.043 (0.037–0.048)] groups relative the MI control group (Figure 7b). N.B. The calculated cardiac Nav1.5 channel gene relative expression in each group represents the average of three replicates for each group.

## 3. Discussion

Azithromycin, clarithromycin, and erythromycin are broadly prescribed macrolides that have been associated with an augmented risk of cardiac arrhythmias and sudden cardiac death [14]. However, “torsade de pointes” has been rarely found with one of the macrolide antibiotics, azithromycin [15,16]. Therefore, there are still unknown mechanisms that led to such unusual forms of drug-mediated pro-arrhythmia requiring further investigation.

The current experimental study aims to explore the potential impact of macrolide treatment on the cardiac Na^+^ channel expression and the alterations in the electrophysiological characteristics of normal versus ischemic heart disease through assessing the alterations of serum cardiac enzymes, including LDH and CK-MB and ECG parameters (HR, PR and QT intervals). The study’s findings showed significant cardiac enzyme changes in the three macrolide antibiotic groups (azithromycin, clarithromycin, and erythromycin) when comparing their mean values with the control and AMI groups. Regarding the PR and QT intervals, the results showed that both intervals are significantly prolonged in the clarithromycin and erythromycin when comparing with the negative control group. In contrast, the group of azithromycin showed prolongation in the two intervals without statistical significance. Expression of the Na1.5 (*SCN5A*) channel gene showed a significant reduction in rats treated with the three studied macrolides with/without MI induction. 

This experimental study demonstrates a possible explanation of the electrophysiological and biochemical alterations of the cardiac Na^+^ channels following three macrolide antibiotics administration in healthy and acute MI rats model, considering that cardiac ion channels have an important role in regulating the cardiac rhythm [17].

As demonstrated in the study results, acute MI rats exhibited ECG alterations, particularly PR and QT intervals with a high serum concentration of serum LDH, CK, and CK-MB. Various prior studies have recruited the isoproterenol hydrochloride inducing similar characteristics in the rats [18]. It was found that macrolides can lead to QT prolongation in rats [12]. Ohtani et al. [19] found that QT prolongation in rats who received macrolides occurred in a dose-dependent manner. It was also found that the most effective antibiotic is erythromycin, then clarithromycin and roxithromycin, and finally, the least likely antibiotic to cause cardiac arrhythmias is azithromycin.

Furthermore, a study found that erythromycin, clarithromycin, and azithromycin prolonged the QT interval, but after lowering potassium concentrations, erythromycin and clarithromycin led to early afterdepolarizations (EADs) and torsade de points, whereas azithromycin did not [20]. In this study, ECG waves and intervals have been more altered by using the same doses of the three macrolide antibiotics demonstrating cardiac arrhythmia in most acute MI rats. PR and QT intervals are significantly prolonged in the clarithromycin and erythromycin, whereas azithromycin showed prolongation in the two intervals without statistical significance.

Irregular and rapid beats of the atrial heart chambers induced with atrial fibrillation reduce the concentration of the ionized transitory outward Ito currents [21] L-type calcium currents (Ca^+2^) and sodium currents (Na^+^), promoting cardiac arrhythmia [22]. Atrial rate more than 400 bpm for a long period induced by atrial fibrillation accordingly leads to substantial alterations in the ion channels function (reduction in transitory outward Ito, Ca^+2^, and Na^+^ densities).

In contrast, the persistent alteration in Na^+^ affects the heart’s rhythm, which is increased by the mutational characteristics in the gene of *SCN5A* that clears the cardiac tetrodotoxin-insensitive Na^+^ currents in individuals experiencing a congenital prolonged QTc disorder [23]. A reduced heart rate and occurring systolic episodes were shown in the individuals who carry the mutational *SCN5A* cognate related to a fatal arrhythmogenic disorder with prolonged QT known as Brugada syndrome [24]. As well as the prolonged QTS by mutations results in functional impairment. Trials imitating a prolonged QTS and restricted clinical trials presenting Na^+^ blockers may be suggested as a particular treatment for deficiencies related to persistent interior Na^+^ currents [25]. 

A previous study showed that azithromycin led to an undescribed type of drug-induced arrhythmia, demonstrated polymorphic ventricular tachycardia without ECG parameters or structural abnormalities, specifically QT interval prolongation, which was found normal [26]. Yang et al. [27] showed that chronic exposure to azithromycin led to polymorph ventricular tachycardia without QT prolongation through altering the cardiac Na^+^ current and promoting loading of intracellular Na^+^ in the human embryonic kidney (HEK 293) and Chinese hamster ovary cells where human Na^+^ channels (*SCN5A*) heterologously expressed. Notably, they used two doses of 50 and 100 mg/kg in acute dosing, and this dose was enough to get the QT prolongation and the other suppressive manifestations on the ECG. Importantly, we used a much longer duration in our experiment, and it was expected to notice much more QT prolongation on the ECG and reduced conductance on Nav1.5. Differently, Fujikawa et al. demonstrated that macrolide antibiotics suppress the airway epithelial sodium channels in vitro [28]

Inherited arrhythmia syndromes, including sudden infant death syndrome, have been linked with mutations in *SCN1B*, encoding the Na^+^ channel β1 subunit. *SCN1B* deletion in mice hearts demonstrated increased peak INa, delayed after-depolarizations, and polymorphic ventricular tachycardia [29]. Clancy et al. [30] showed a mutation in the *SCN5A* gene raised Na^+^ influx by augmenting the Na^+^ channel window current and associated with ventricular tachycardia. 

We observed higher mortality % in MI rats than healthy rats when they received chronic macrolide doses. Although mortality may be developed due to other body toxicities, the great difference between the healthy rats & rats with MI indicates that cardiotoxicity may be the main reason for the predisposing to death. In the current study, we focused on cardiotoxicity as a main toxic effect for macrolides in rats. Indeed, it was reported that when azithromycin 200 mg/kg was given to mice for ten days, the concentration in the cardiomyocytes may reach 30–220 fold that of plasma concentration [31]. The same finding was confirmed in non-myocardial tissues, though the serum level falls to trough level within a day [32]. These findings support the view that cardiotoxicity may be the main cause of death in the current model of rat MI. Nevertheless, we can also highlight that further studies are warranted to determine other organ toxicities. 

## 4. Materials and Methods

### 4.1. Antibiotic Preparations 

Azithromycin in the form of Zithromax Suspension (Pfizer Pharmaceutical Company, Cairo, Egypt), clarithromycin (Klarimix oral suspension, Sigma Pharmaceutical Company, Quesna, Egypt), and erythromycin (Erythrocin oral suspension, AlQahira Company for Abbott Lab, Cairo, Egypt) were used.

### 4.2. Experimental Animals 

Eighty male Wistar albino rats with a body weight range (80–126 g) were used in this study. Rats were housed in clean stainless-steel cages under fixed laboratory settings and a normal light-dark cycle. Water and food were provided *ad libitum*. Rats were acclimatized to the animal house conditions. The protocol of the current experiment was approved by the institutional research ethics committee at the Faculty of Pharmacy, Suez Canal University, and performed according to the guidelines of the care and use of laboratory animals issued by the National Institutes of Health (NIH Publications No. 8023, revised 1978). 

### 4.3. Induction of Experimental Myocardial Infarction by Isoproterenol

Isoproterenol HCL (ISOP-HCL) in a powdered form was purchased from Sigma Aldrich Company (MO, USA) and dissolved in a sterile saline solution immediately before use each day. ISOP-HCL was employed to induce experimental MI in rats and was administered in two subcutaneous doses (85 mg/kg per 24 h) following a previously reported schedule [33,34,35].

### 4.4. Dose Justification for Macrolide Antibiotics

The three macrolide antibiotics were used in 100 mg/kg doses in rats. When the authors translate these doses to the equivalent human doses according to the equation designed by Reagan-Shaw et al. depending on the body surface area, we found that the human equivalent of murine doses of 100 mg/kg is 973 mg for an adult human (60 kg). Since these medications can be used in a maximum daily dose equals 1 g for Azithr (1000 mg), clarithr (1500 mg), or erythromycin (1–4 g per day). Hence, the currently utilized doses were within the safe range recommended for humans

Previous rat studies support these calculations. For example, the rat dose of Azith was reported to be 100 mg/kg [36]. Further, Sassa et al. reported rat oral doses of Clarith equal 2 mg/kg of body weight; hence an average weight of 100 g rat will receive 100 mg of Clarith [37].

### 4.5. Study Design

Rats were allocated into two experiments: each one consisted of 40 rats distributed equally into four groups as follows:

Experiment 1: Healthy rats

Group 1: Vehicle (Saline) group, Group 2: Azithromycin (100 mg/kg) group, Group 3: Clarithromycin (100 mg/kg), Group 4: Erythromycin (100 mg/kg) group.

Experiment 2: Rats with MI

Group 5: MI control group, Group 6: MI + Azithromycin (100 mg/kg) group, Group 7: MI + Clarithromycin (100 mg/kg), Group 8: MI + Erythromycin (100 mg/kg) group.

In general, ISOP-HCL was injected during the first two days of the experiment, and drug administration by gastric gavage started from the second week of the experiment and continued for seven weeks (Table 2). After finishing the antibiotic assigned courses, survival % in each experimental group was calculated as (the number of surviving rats/10) × 100 and then ECG monitoring was done under slight ether anesthesia. 

### 4.6. Electrocardiography

After completing the drug schedule, research Biopac data acquisition mp150 device (BIOPAC Systems, Inc.; Goleta, CA, USA) was used for ECG recording. Next to light ether anesthesia, rats were put on a panel while their legs were restrained following a previous method. Some ECG parameters were determined from ECG traces; HR (in beats per minute), QT interval (in milliseconds), and RR Interval = 60/H.R. [38]. 

### 4.7. Animal Scarification and Specimen Collection

Blood samples were collected from rats by cardiac puncture under anesthesia. Rats were then sacrificed by cervical dislocation, and then the hearts were dissected and washed out of the blood. Blood samples were centrifuged at 4000× *g* for 13 min. Serum samples were isolated and kept at −80 °C until used for the biochemical assays. 

Cardiac tissue samples were dissected from the left ventricles and fixed in paraformaldehyde solution overnight. Samples were then washed in tap water, followed by serial dilutions of alcohol. Then, the specimens were embedded in paraffin wax. Paraffin blocks were sectioned at 4-μm and collected on glass slides for subsequent staining with hematoxylin and eosin (HE) [39] and Masson’s trichrome staining to show the degree of myocardial fibrotic changes.

A histopathologic score was assigned for each cardiac specimen according to the previously reported criteria: the inflammatory cell infiltrates degree, striation loss, cardiomyocyte degeneration, and inter-muscular hemorrhage and edema). Scoring of the listed findings was done according to the intensity and frequency as (0) absent, (1) weak to low, (2) mild to moderate, and (3) high to frequent, and the total score was calculated [18,40]. Masson’s trichrome staining was assessed by ImageJ software (NIH, Bethesda, Rockville, MD, USA; Version 1.53) to measure the area of fibrosis in 2 fields/specimens, and the average was taken for each rat. 

### 4.8. Determination of Serum LDH and CK-MB 

Serum lactate dehydrogenase (LDH) activity was determined by an enzymatic assay kit according to a previous method [24]. Also, the activity of serum creatine kinase-MB (CK-MB) isozyme was measured by a documented immune-inhibition method. The kits were supplied by the National Bio Lab in (Giza, Egypt) according to the procedures listed by the manufacturer using a Shimadzu spectrophotometer (UV1601-PC, Kyoto, Japan) [39,41]. 

### 4.9. Quantitative Real-Time Reverse Transcription-Polymerase Chain Reaction (RT-PCR) Analysis of Cardiac Sodium Nav1.5 Ion Channel Gene Expression

Cardiac tissue total RNA was extracted following the RNeasy FFPE Kit (ID: 73504, Qiagen, Hilden, Germany) manufacturer’s instructions. The DNase and DNase booster buffer were applied during the work for optimized removal of genomic DNA contamination. The concentration and purity of the extracted RNA were checked by the NanoDrop ND-1000 spectrophotometer (NanoDrop Tech., Inc. Wilmington, DE, USA). The reverse transcription step was carried out using the “High-Capacity cDNA Reverse Transcription Kit (Applied Biosystems, P/N 4368814)”. For each sample, a reaction mixture (15 μL) contains the extracted RNA (5 μL), 100 mM of each deoxynucleotide triphosphate (0.15 μL), MultiScribe^®^ reverse transcriptase (50 U/μL), RT buffer (10×) (1.5 μL), RNase inhibitor (20 U/mL), SCN5A TaqMan^®^ specific primers (3 μL of each), and nuclease-free water (4.16 μL), was incubated in the “T-Professional Basic, Biometra PCR System (Biometra, Göttingen, Germany)” for 30 min at 16 °C, then 30 min at 42 °C, and 5 min at 85 °C, with subsequent holding stage at 4 °C. Non-template and non-reverse transcriptase negative controls were applied in each experiment to ensure the absence of genomic DNA contaminations [42].

The *SCN5A* gene (ID: 25665) expression was quantified using Rat TaqMan^®^ gene expression assay (ID: Rn00565502_m1). The predesigned assay contains gene-specific primers/Probs (Applied Biosystems, Foster City, CA, USA). TaqMan^®^ endogenous control assay for *GAPDH* (ID: Rn01775763_g1, Applied Biosystems, Foster City, CA, USA) was utilized to correct variation in RNA loading potential (data normalization). For each tissue sample, five PCR reactions were done in the final volumes of 20 µL, including the cDNA (1.33 µL), 2× TaqMan^®^ Universal PCR Master Mix and TaqMan^®^ assay (1 µL). The appropriate negative controls that do not include either the template or reverse transcriptase enzyme were run with each plate. The PCR was performed on StepOne™ Real-Time PCR System (Applied Biosystems), and the PCR program was done as reported previously [43]. The Minimum Information for Publication of Quantitative Real-Time PCR Experiments (MIQE) guidelines were followed during the PCR work, including running the appropriate negative controls with each PCR run, the blind evaluation to the study sub-groups status, and application of three technical replicates for each sample with the calculation of the mean values [44]. The relative expression of the specified study gene in each group was calculated using the equation 2^−ΔΔCq^, where “ΔΔC_q_ (delta delta quantitative cycle) = (C_q_ *SCN5A* − C_q_ *GAPDH*)_MI group_ − (C_q_ *SCN5A* − C_q_ *GAPDH*)_Healthy control_” [13].

### 4.10. Statistical Analysis

Data confirmed to be in a normal distribution are presented as the mean ± SD and were evaluated using 1-way analysis of variance (ANOVA) and Bonferroni’s post-hoc test. Comparisons were made by the SPSS program and GraphPad Prism (ISIH software, USA) version 6 at *p*-value < 0.05. Further, the histopathologic scoring data were presented as medians with maximum and minimum values, while statistical analysis was done using the Kruskal Wallis test followed by Dunn’s test. The pair-wise comparison between the vehicle group in healthy rats and the MI control group was done using student’s *t*-test. Differently, the survival % was categorized as qualitative data and was analyzed using the Chi-square test. The 0.05 level of probability was set as the accepted level of significance.

## 5. Conclusions

This experimental study was designed to investigate the impact of chronic use of three macrolide antibiotics and estimate their adverse effects on MI rats compared to healthy rats. Our results proved that treating AMI rats’ with any of the three macrolide antibiotics elevated serum LDH and CK-MB and changed ECG parameters causing cardiac arrhythmias associated with potential *SCN5A* gene expression downregulation. Hence, comprehensive studies are needed to either support or decline this view in humans.

## Figures and Tables

**Table 1:** The effect of three macrolide antibiotics on the ECG parameters in healthy rats and MI model rats.

Groups	PR Interval (ms)	QT Interval (ms)	HR (Beat/min)
Vehicle	40.1 ± 1.79	88.6 ± 2.37	369.3 ± 8.65
Azith	44.3 ± 1.50 *	93.8 ± 1.05 *	379.3 ± 2.56 *
Clarith	43.9 ± 2.2 *	93.6 ± 137 *	375.3 ± 1.44 *
Eryth	44.2 ± 1.89 *	94.6 ± 1.65 *	381.1 ± 2.34 *
MI+ Vehicle	46.4 ± 1.90	94.2 ± 2.15	388.1 ± 8.21
MI + Azith	48.8 ± 5.22	95.6 ± 3.37	389.9 ± 80.69
MI + Clarith	49.6 ± 2.32 ^a^	99.3 ± 2.91 ^a^	425.5 ± 45.63 ^a^
MI + Eryth	50.1 ± 3.3 ^a^	98.2 ± 1.71 ^a^	426.6 ± 36.33 ^a^

Data are mean ± standard deviation, * Different from the vehicle group, ^a^ Different from the MI + Azith group at *p*-value less than 0.05, (*n* = 4–6).

**Table 2:** A diagram demonstrating the course of the experiment.

Weeks	1	2	9	10
	Healthy	Vehicle or Oral Macrolides	Functional assessment	Blood tests & Histology
	MI	Vehicle or Oral Macrolides	Functional assessment	Blood tests & Histology

## Data Availability

Data are available from the authors upon request.
